# Critical Data-Based Incremental Cooperative Communication for Wireless Body Area Network

**DOI:** 10.3390/s18113661

**Published:** 2018-10-28

**Authors:** Hameed Al-Mishmish, Ahmed Alkhayyat, Hasliza A. Rahim, Dalal A. Hammood, R. Badlishah Ahmad, Qammer H. Abbasi

**Affiliations:** 1 Department of Electrical and Communication Engineering, Cankaya University, 06530 Ankara, Turkey; ahmedalkhayyat85@gmail.com; 2 Department of Computer Technical Engineering, College of Technical Engineering, The Islamic University, 54001 Najaf, Iraq; 3 Bio Electromagnetic Research Group (BioEM), School of Computer and Communication Engineering University Malaysia Perlis (UniMAP), Pauh Putra, Arau, Perlis 02600, Malaysia; haslizarahim@unimap.edu.my (H.A.R.); dalal@studentmail.unimap.edu.my (D.A.H.); 4 Faculty of Informatics and Computing, Universiti Sultan Zainal Abidin (UniSZA), Kuala Terengganu 21300, Malaysia; badli@unisza.edu.my; 5 School of Engineering, University of Glasgow, G12 8QQ Glasgow, UK; Qammer.Abbasi@glasgow.ac.uk

**Keywords:** wireless body area network, critical data index, incremental cooperative communication, outage and successful probability, end-to-end delay, duty cycle, average power transmission

## Abstract

Wireless Body Area Networks (WBANs) are single-hop network systems, where sensors gather the body’s vital signs and send them directly to master nodes (MNs). The sensors are distributed in or on the body. Therefore, body posture, clothing, muscle movement, body temperature, and climatic conditions generally influence the quality of the wireless link between sensors and the destination. Hence, in some cases, single hop transmission (‘direct transmission’) is not sufficient to deliver the signals to the destination. Therefore, we propose an emergency-based cooperative communication protocol for WBAN, named Critical Data-based Incremental Cooperative Communication (CD-ICC), based on the IEEE 802.15.6 CSMA standard but assuming a lognormal shadowing channel model. In this paper, a complete study of a system model is inspected in the terms of the channel path loss, the successful transmission probability, and the outage probability. Then a mathematical model is derived for the proposed protocol, end-to-end delay, duty cycle, and average power consumption. A new back-off time is proposed within CD-ICC, which ensures the best relays cooperate in a distributed manner. The design objective of the CD-ICC is to reduce the end-to-end delay, the duty cycle, and the average power transmission. The simulation and numerical results presented here show that, under general conditions, CD-ICC can enhance network performance compared to direct transmission mode (DTM) IEEE 802.15.6 CSMA and benchmarking. To this end, we have shown that the power saving when using CD-ICC is 37.5% with respect to DTM IEEE 802.15.6 CSMA and 10% with respect to MI-ICC.

## 1. Introduction

WBANs are the communication networks of sensor nodes (and/or actuators) placed on, inside, or around the human body that shows a new generation of the wireless personal area network (WPAN), and introduce several challenges for implementation. The sensor nodes in WBANs are small and embedded with finite source compared to devices in the traditional wireless sensor networks (WSN). Finite source make a limitation on the energy spent by sensor nodes in sensing, processing, storing and delivering the data [1,2,3,4]. 

The end-to-end (e2e) delay, the duty cycle and the average power transmission are the key factors to determine the overall performance of a WBAN. The most suitable layers to address the aforementioned factors are data link layer, such as medium access control (MAC) protocol and physical layer (such as virtual diversity technique) [5,6,7]. MAC protocol is controlling and organizing the sensor nodes access to the wireless shared medium. MAC protocol is an essential protocol which consider the basis for getting Quality of Service (QoS), high data rate and higher power saving in any wireless networks. In addition, the MAC protocol is preventing collisions and concurrent sending while conserving data rate, reduce e2e delay, and enhanced the reliability [8,9,10,11]. 

The diversity technique is the method to combat the effect of the of the wireless channel fading, diversity can be achieved through either embedded the sensor node with multiple antennas or through using the cooperative communication (CC) [12,13]. Various type of the CCs is considered in WBAN to improve their performance in the term of power transmission, reliability, and the e2e delay. Where, in the traditional cooperative communication (TCC), a source sends data to a one of the on body intermediate node, then intermediate node(s) (relay(s)) retransmit what was sent by the source to the destination [14]. However, such cooperative communication utilized extra sub-channels/time slots to transmit single data from the source to the destination, which increases the delay, and reduce the bandwidth efficiency of the wireless communication [15,16]. Therefore, to solve the aforementioned problem of the TCC, an incremental cooperative communication (ICC) is utilized. In such, the intermediate node does not participate in cooperation until the destination does not receive what was sent by the source correctly [17]. The TCC has been widely considered in the literature for WBAN systems [18,19,20,21,22,23,24,25,26], however, in this paper, only the ICC is surveyed. 

Deepak et al. [27] investigated the energy efficiency (EE) of incremental cooperative communication (ICC) in WBANs. They also provided an analytical model for the EE of DTM and CCs and considered the effect of packet error rate (PER) on both systems. The optimisation of packet size was also taken into account. Paul et al. [28] and Yousaf et al. [29] optimised the packet size to maximise the EE in IEEE 802.15.6, considering ultra-wideband, where packet size optimisation was done for both DTM and ICC systems. Liao et al. [30] reduced the energy consumption, and prolonged the network lifetime, of in-body sensor nodes by using an ICC protocol. They maintained a flexible QoS and suggested a new in-to-out body path loss (PL) model. Estevez et al. [31] proposed a novel cooperative energy harvesting (CEH)-MAC model, that adapted its operation to the energy harvesting (EH) conditions. Their proposed protocol exploits the EH information in order to set an idle time that allows the relay nodes to charge their batteries and complete the cooperation phase successfully. They have improved EE, e2e delay, and network throughput. Yousaf et al. [32] investigated and analysed ICC for WBANs with different numbers of relays where EE and PER were inspected for various scenarios. Also, a new ICC with three-stage relaying of data is proposed (the so-called ‘Enhanced Incremental Cooperative Critical data transmission in Emergencies for Static WBANs’ (EInCo-CEStat)) where the proposed protocol enhances the EE and PER compared to existing work. Prakash et al. [33] proposed a Linear Acceleration based Transmission Power Decision Control (LA-TPDC) algorithm where energy consumption, signal-to-noise ratio (SNR), bit error rate (BER), and PER were evaluated for all participating nodes. Liao et al. [34] proposed mutual information-based incremental cooperative communication (MI-ICC) protocol, where several on-body relay nodes and one coordinator were attached to the patient’s clothes. MI-ICC took into account the critical data, while the normal data does not get transmitted to the destination. It achieved better performance in comparison to the scheme using two relays, with the residual energy and network lifetime taken into account and improved. 

In what follows, the drawbacks and limitations of [27,28,29,30,31,32,33,34] are shown in Table 1 and can be summarised as follows: MAC protocol was not considered (such as IEEE 802.15.6), e2e delay was not analysed, the best relay node selection was not considered, the duty cycle was not analysed, and the average power transmission was not studied, and the nature of the gathered data was not taken into account. 

To address the aforementioned issues and facilitate cooperative communication in WBAN, we propose a novel Critical-Data Incremental Cooperative Communication protocol based on the IEEE 802.15.6 CSMA policy. The contributions of this work are summarised as follows:

1. A MAC protocol for the CD-ICC is proposed to coordinate the sensor to act as relay to carry out the retransmission process.
2. A new back-off time is proposed to achieve the selection of the best relay, where only the sensor that is nearest to the source can participate in cooperation. In addition, the back-off time accelerates the access of the selected best relay to the shared medium.
3. The gathered data natures have been considered. Where the critical data is transmitted over ICC, while the normal data is transmitted over DTM. It is meant that CD-ICC protocol supports multiple traffics.
4. The e2e delay, duty cycle and average power transmission of CD-ICC are mathematically modelled and analysed.
5. We show that the proposed protocol can reduce the e2e delay and the duty cycle and can enhance power saving of the WBAN compared to the existing work and DTM under IEEE 802.15.6 CSMA policy.

The rest of the paper is organized as follows: system model and architecture is presented in Section 2. Section 3 describes and investigate the wireless link and successful transmission probability under lognormal shadowing model. Then, modeling and formulating of CD-ICC in details has been described in Section 4. In Section 5, e2e delay, duty cycle and average power transmission of CD-ICC are investigated, formulated and analyzed. Simulation and numerical results are addressed in Section 6. Finally, Section 7 draws the conclusion and future work.

## 2. System Model and Architecture

**Figure 1:**
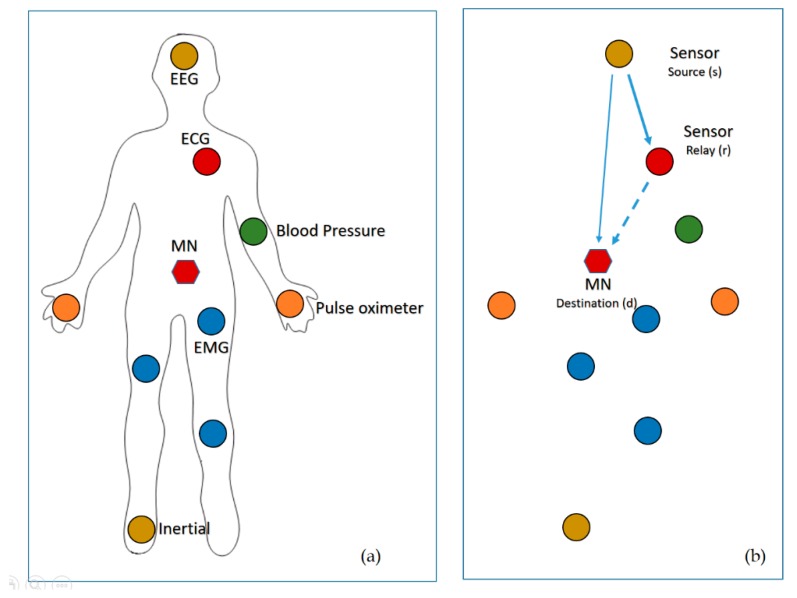
(**a**) Network architecture of WBAN; (**b**) Cooperative communication in WBAN.

Figure 1a shows an example of a WBAN system. There are many sensors uniformly distributed around the body to monitor the patient’s health, and each sensor gathers data and sends it to the MN. In a WBAN that is based on the single-hop star topology, all the sensors send their data directly to the MN. The MN then directs the data to the monitor node, which either analyses it, or forwards it over the internet to the hospital or doctors. 

In WBANs, it should consider a number of physical environments, due to the networks are configured on/in the body. Where, the sensors attached to the body are mobile owing to numerous body movements. Therefore, the distances between the sensors and MN are varying. Some sensors may have a large distance or weak link to the MN, thus transporting data sufficiently to the MN in a single-hop is difficult. 

The CC is considered one of the best solutions to overcome the aforementioned problem, i.e., single hop transmission. Various CC modes are widely inspected in the literature. The ICC is considered in this work and it is summarized as follow: if the MN (or destination) received the data correctly from the source sensor (S) based on frame check sequence (FCS), then it sends an acknowledgment (ACK) to the S and the relay sensor (R) drop what received from S. Otherwise, it sends a negative acknowledgment (NACK) that allows the R retransmit what was received from the S, but MN drop what received from the S, see Figure 1b. In what follow, the distance from S to MN, S to R and R to MN are denoted as $d_{sd}$, $d_{sr}$ and $d_{rd}$, respectively, and we denoted link between sensor and MN as S−D, link between source and relay sensor as S−R and the link between relay sensor to MN as R−D.

## 3. Link and Successful Transmission Probability Analysis 

In this section, the link analysis and successful transmission probability under lognormal channel model are described. The received power at any given distance can be expressed as [35]:

(1) $$P\left(d_{ij}\right)={\left(\frac{d_{ij}}{d_{o}}\right)}^{-\rho }$$

where, $d_{o}$ is a reference distance and ρ is the path-loss exponent. $d_{ij}$ is the distance between node i and j. Afterwards, according to the lognormal channel model, the received power can be expressed as [35]:

(2) $$10\log_{10}\left(P\left(d_{ij}\right)\right)=10\log_{10}\left(P_{a}\left(d_{ij}\right)\right)+\sigma_{ij}^{2}$$

For the sake of the simplicity, we normalized variables as follows: first, let defined G as the maximum distance where the received power $P_{a}\left(d_{ij}\right)$ is equal to $P={\left(\frac{G}{d_{o}}\right)}^{-\rho }$. Then, by dividing powers by *P* and with help of (2), the received power under lognormal channel model can be expressed as:

$$10\log_{10}\left(\frac{P\left(d_{ij}\right)}{P}\right)=10\log_{10}\left({\left(\frac{d_{ij}}{G}\right)}^{-\rho }\right)+\sigma_{ij}^{2},$$

then, this yields:

(3) $$10\log_{10}\left(P^{n}\left(d_{ij}\right)\right)=10\log_{10}\left({\left(d_{ij}^{n}\right)}^{-\rho }\right)+\sigma_{ij}^{2}.$$

where, $d_{ij}^{n}≜\frac{d_{ij}}{G}$ is the normalized distance and $P^{n}\left(d_{ij}\right)≜\frac{P\left(d_{ij}\right)}{P}$ is the normalized power. It is shown that the l0garithm of a normalized power has a normal distribution with the mean $10\log_{10}\left(d_{ij}^{n}\right)$ and the variance $\sigma_{ij}^{2}$. The condition for correct reception of signals at normalized distance $d_{ij}^{n}$ is that the normalized power at this distance is more than ‘1’ or zero dB. The probability of successful reception at node j due to transmission of node i can be expressed as:

(4) $$\begin{array}{l} P_{ij}^{s}=P\left[10\log_{10}\left(P^{n}\left(d_{ij}\right)\right)>0\right]\\ \;=\frac{1}{\sqrt{2\pi }\sigma_{ij}}\;\overset{\infty }{\underset{0}{\int }}\exp\left(-\frac{{\left(r-10\log_{10}\left({\left(d_{ij}^{n}\right)}^{-\rho }\right)\right)}^{2}}{2\sigma_{ij}^{2}}\right)dr \end{array}$$

this yields:

(5) $$P_{ij}^{s}=0.5\;erfc\left(\frac{\omega }{U_{ij}}\right)$$

where erfc(x) (=1−erf (x)) is complementary error function, $U_{ij}=\sigma_{ij}/\log d_{ij}^{n}$ and $\omega =10\rho /\sqrt{2}\log10$.

## 4. Critical Data-Based Incremental Cooperative Communication (CD-ICC)

### 4.1. Proposed Protocol Description 

In this paper, we propose an emergency-based cooperative communication for WBAN, named Critical Data-based Incremental Cooperative Communication. The proposed protocol works in a cooperative fashion when considering critical data. The CD-ICC has two events which can be summarised as follows:

1. The first event is the Critical Data Event (denoted as X) which is occurs when data gathered by the sensor is critical and must be transmitted to the destination efficiently. In such cases, critical data is delivered to the destination utilising ICC.
2. The second event is the Normal Data Event (denoted as Y) which occurs when data gathered by the sensor isn’t critical and it can be transmitted directly to the destination.

### 4.2. Formulation of the CD-ICC 

As described earlier, the CD-ICC comprised from two events and is mathematically expressed as:

(6) $$P_{CD-ICC}=\underset{Critical\;data\;event}{\underbrace{P\left(X\right)}}+\underset{Normal\;data\;event}{\underbrace{P\left(Y\right)\;}}$$

where, P(X) is the probability of the gathered data were critical. The critical data delivered to destination utilizing ICC and is mathematically expressed as:

(7) $$P\left(X\right)=P\left(P\left(\xi \geq \xi_{thd}\right),P_{ICC}^{s}\right)$$

where, the $P\left(\xi \geq \xi_{thd}\right)$ is the probability of critical data index ξ greater than threshold value $\xi_{thd}$, and $P_{ICC}^{s}$ the successful transmission probability of the ICC. In the (7), events are independent, then the P(X) can be written as:

(8) $$P\left(X\right)=P_{\xi }\left(\xi_{thd}\right)P_{ICC}^{s}$$

The $P_{ICC}^{s}$ can be expressed as:

(9) $$P_{ICC}^{s}=P_{sd}^{s}+\left(1-P_{sd}^{s}\right)P_{sr}^{s}P_{rd}^{s}$$

where, the term $P_{sd}^{s}$ represent S−D link isn’t in the outage, while $\left(1-P_{sd}^{s}\right)$ represent the S−D link in the outage, and the terms $P_{sr}^{s}$ and $P_{rd}^{s}$ represent S−R and R−D links are not in the outage. Afterwards, and with help of (5), we obtain $P_{ICC}^{s}$ as:

(10) $$P_{ICC}^{s}=0.5erfc\left(\frac{\omega }{U_{sd}}\right)+0.25erfc\left(\frac{\omega }{U_{sr}}\right)erfc\left(\frac{\omega }{U_{rd}}\right)\left(1-0.5erfc\left(\frac{\omega }{U_{sd}}\right)\right)$$

where, ξ is normal random variable with zero mean and unity variance, hence the probability that the critical data index ξ greater than threshold value $\xi_{thd}$ is given as:

(11) $$P_{\xi }\left(\xi_{thd}\right)=P\left(\xi \geq \xi_{thd}\right)=0.5\overset{\infty }{\underset{\xi_{thd}}{\int }}g\left(y\right)dy$$

where, g(y) is given as:

(12) $$g\left(y\right)=\frac{1}{\sqrt{2\pi }}\exp\left(-\frac{y^{2}}{2}\right)$$

insert (12) in (11), we obtain (11) as:

(13) $$P_{\xi }\left(\xi_{thd}\right)=0.5\overset{\infty }{\underset{\xi_{thd}}{\int }}\frac{1}{\sqrt{2\pi }}\exp\left(-\frac{y^{2}}{2}\right)dy$$

solving the integral of (13), we obtain (13) as:

(14) $$P_{\xi }\left(\xi_{thd}\right)=erfc\left(\xi_{thd}\right)$$

where, $\xi_{thd}$ is threshold that gathered data is critical, and it expressed as:

(15) $$\xi_{thd}=\left|\frac{\xi_{min}-\xi_{max}}{\xi_{max}}\right|$$

A parameter called the critical data index threshold, $\xi_{thd}$, determines the degree of criticality. $\xi_{max}$ is the maximum critical data index and is equal to 7, while $\xi_{min}$ is the minimum critical data index and can vary between 0 and 7. $\xi_{min}$ depends on the gathered data from the human body, and if the data is critical, then $\xi_{min}$ takes a high value, and vice versa. Table 2 show the probability of the critical data index with different values of $\xi_{min}$.

It is clear that, as the $\xi_{thd}$ is high, the probability of the critical data index is low and vice versa. Inserting (10) and (14) in (8), we obtain P(X) as:

(16) $$\begin{array}{l} P\left(X\right)=erfc\left(\xi_{thd}\right)(0.5erfc\left(\frac{\omega \;}{U_{sd}}\right)\\ \;+0.25erfc\left(\frac{\omega \;}{U_{rd}\;}\right)erfc\left(\frac{\omega \;}{U_{rd}\;}\right)\left(1-0.5erfc\left(\;\frac{\omega \;}{U_{sd}\;}\right)\right)) \end{array}$$

The second term of the (6) represent the event that the gathered data by the sensor were not critical and with help of (5) and (14), the *P*(*Y*) can be expressed as:

(17) $$P\left(Y\right)=\frac{1}{2}\left(1-erfc\left(\xi_{thd}\right)\right)erfc\left(\frac{\omega \;}{U_{sd}}\right)$$

finally, summing up the *P*(*X*) and *P*(*Y*) together, we obtain $P_{CD-ICC}$ as:

(18) $$\begin{array}{l} P_{CD-ICC}\\ =\underset{critical\;data\;event}{\underbrace{erfc\left(\xi_{thd}\right)\left(0.5erfc\left(\frac{\omega \;}{U_{sd}\;}\right)+0.25erfc\left(\frac{\omega \;}{U_{rd}\;}\right)erfc\left(\;\frac{\omega \;}{U_{rd}\;}\right)\left(1-\;0.5\;erfc\left(\;\frac{\omega \;}{U_{sd}\;}\right)\right)\right)}}\;\\ +\underset{normal\;data\;event}{\underbrace{\;0.5\left(\;1-\;erfc\left(\xi_{thd}\right)\right)\;erfc\left(\;\frac{\omega \;}{U_{sd}\;}\right)}}\; \end{array}$$

## 5. Delay and Duty Cycle Analysis of CD-ICC

### 5.1. Delay Analysis of CD-ICC

**Figure 2:**
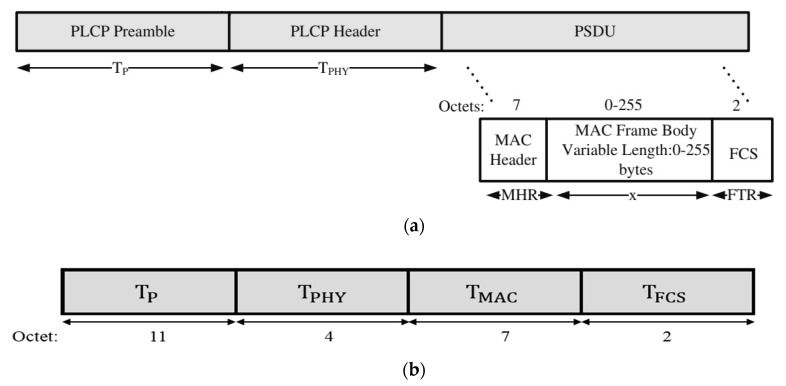
(**a**) IEEE 802.15. Physical Protocol Data Unit frame structure; (**b**) IEEE 802.15. ACK packet.

The average e2e delay of the IEEE 802.15.6 of CD-ICC is evaluated in this subsection. Where, the average e2e delay is defined as the total time required of the medium access delay to transmit data. The average e2e delay includes average contention time due to collision ($T_{C}$), the average successful transmission time with no collision and no fading ($T_{suc}$), and average failure time due to fading but no collision ($T_{fail}$) [36]:

(19) $$T_{e2e}=T_{C}+T_{suc}+T_{fail}$$

the average contention time due to collision can be expressed as:

(20) $$T_{C}=T_{data}+T_{I-ACK}+T_{CW}+2T_{pSIFS}+2T_{\alpha }$$

the time required to transmit data packet (see Figure 2a) can be expressed as:

(21) $$T_{data}=T_{P}+T_{PHY}+T_{MAC}+T_{BODY}+T_{FCS}.$$

the transmissi0n time required for I−ACK (see Figure 2b) can be expressed as:

(22) $$T_{I-ACK}=T_{P}+T_{PHY}+T_{MAC}+T_{FCS}.$$

where $T_{CW}$ average back0ff time and is expressed as:

(23) $$T_{CW}=T_{s}\;CW$$

Average successful transmission time with no collision and no fading can be expressed as:

(24) $$T_{suc}=P_{\xi \;}\left(\xi_{thd}\right)\;\left(T_{act}^{sd}\;P_{sd}^{s}+\;\left(T_{act}^{sd}+T_{act}^{rd}\right)\;\left(1-\;P_{sd}^{s}\;\right)\;P_{sr}^{s}\;P_{rd}^{s}\right)+\left(1-\;P_{\xi \;}\left(\xi_{thd}\right)\right)\;P_{sd}^{s}\;T_{act}^{sd}$$

where $T_{act}^{sd}\;$is the RF activity time of S−D link, $T_{act}^{sd}=\;T_{C}+T_{on}$. Re-write (24) as:

(25) $$T_{suc}=P_{\xi \;}\left(\xi_{thd}\right)\;\left(T_{act}^{sd}+T_{act}^{rd}\right)\;\left(\;1-\;P_{sd}^{s}\;\right)\;P_{sr}^{s}\;P_{rd}^{s}+\;P_{sd}^{s}\;T_{act}^{sd}$$

Equation (24) comprises two terms. The first term is e2e required time of the transmission when the gathered data by the sensor are critical, delivered through ICC. The second term is e2e required time of the transmission when the gathered data by the sensor are not critical, delivered over DTM. 

It is clear from (24), as the value of $P_{\xi }\left(\xi_{thd}\right)$ approaches one, either $T_{act}^{sd}$ is the required time to transmit the data or $T_{act}^{sd}+T_{act}^{rd}$ is the required time to transmit the data to the destination. On the other hand, as the value of $P_{\xi }\left(\xi_{thd}\right)$ approaches zero, then $T_{act}^{sd}$ is the required time to transmit the data to the destination. $T_{act}^{rd}$ is the RF activity time of the R−D link and can be expressed as:

(26) $$T_{act}^{rd}=T_{on}^{rd}+T_{CW}^{rd,*}+T_{data}^{rd}+T_{ACK}^{rd}+2T_{pSIFS}^{rd}+2T_{\alpha }^{rd}.$$

In this paper, we propose a new $T_{CW}^{rd}$ expressed as follows:

(27) $$T_{CW}^{CD-ICC}=\lceil T_{s}\;\frac{CW_{max}}{CW_{min}}\left|\left(\frac{d_{eq}\;}{2^{\rho -2}+d_{eq}^{-1}\;}\;\right)\sqrt[{\rho -1}]{d_{eq}}+\left(\rho -4\right)\;\right|\rceil \mu s$$

where $CW_{min}$ and $CW_{max}$ are the minimum contention window and maximum contention window size, respectively. $d_{eq}$ is expressed as ${\left(d_{sr}+d_{rd}\right)}^{-\rho }$. The objective of the proposed back-off time is to make sure that the best relay sensors can participate in cooperation, and that the best relay sensor can access the channel first. However, when the relay sensor is willing to help the source, it may have a greater delay due to (27). Hence, each relay(s) will select the $T_{CW}^{*}$ according to:

(28) $$T_{CW}^{rd,*}=\{\begin{array}{c} CW\;T_{s}\;for\;T_{CW}^{CD-ICC}>T_{CW}\\ T_{CW}^{CD-ICC}\;for\;T_{CW}^{CD-ICC}\leq T_{CW} \end{array}$$

As is clear from (28), the intermediate sensor that overheard the transmitted packet calculates the proposed back-off time as shown in (27). Then, $T_{CW}^{CD-ICC}>T_{CW}$ implies an average back-off time of the relay node (intermediate) is less than the proposed average back-off time (27), and the node cannot participate in cooperation. On the other hand, if $T_{CW}^{CD-ICC}\leq T_{CW}$, this implies the sensors whose overheard the transmitted packet are near to the source, such sensor utilised proposed back-off time shown in (27). Finally, the average failure time due to fading but no collision is expressed as:

(29) $$T_{fail}=(1-P_{sd}^{s})\;\left(1-P_{sr}^{s}\right)P_{rd}^{s}\;T_{act}^{sd}+\left(T_{act}^{sd}+T_{act}^{rd}\right)\;(1-P_{sd}^{s})\;\left(1-P_{rd}^{s}\right)P_{sr}^{s}\;+(1-P_{sd}^{s})\;\left(1-P_{sr}^{s}\right)\;(1-P_{rd}^{s})\;T_{act}^{sd}$$

It is clear from (29), the first term corresponds to the events when the S−D, and S−R links in the outage, while R−D link not in the outage. The second term corresponds to the events when the S−D, and R−D links in the outage, while S−R link not in the outage. The last term is corresponds to the events when the S−D, S−R and S−D links in the outage. In (29), we did not include the $P_{\xi }\left(\xi_{thd}\right)$, because channel fading do not affected by the nature of the data, i.e., whether it is critical or not.

The transmission rate of the PHY, MAC headers and payload are depending on the channel condition between nodes [37]. Where, the $R_{ate}$ of the CD-ICC is given as

(30) $$R_{ate}=P_{\xi }\left(\xi_{thd}\right)\;P_{ICC}^{s}\;R+\left(1-P_{\xi }\left(\xi_{thd}\right)\right)\;P_{sd}^{s}\;R$$

where, R is the transmission rate of IEEE 802.15.6 standard and it is 75.9 Kbps for DPSK modulation [38].

CW:
*Contention window*
α: 
*Delay time*
Ts: 
*CSMA slot length*

$T_{P}:$

*preamble time*

$T_{c}:\;$

*Collision time*

$T_{PHY}:$

*physical header time*
$T_{on}$: 
*RF transceiver power-on*

$T_{MAC}:$

*MAC header*

$T_{CW}:$

*Average back-off time*

$T_{BODY}:$

*MAC frame body time*

$T_{data}:$

*Time to transmit a data packet*

$T_{FCS}:$

*frame check sequence time*

$T_{I-ACK}:$

*Time to transmit ACK*

$T_{PSIFS}:$

*Short interframe spacing*


### 5.2. Duty Cycle and Average Power Transmission of CD-ICC in WBAN

In this subsection, we address the duty cycle (DC) and average power transmission of the proposed protocol under CSMA/CA based on IEEE 802.15.6. The average power transmission related directly to the duty cycle. DC is defined as the ratio of the time required to transmit a packet successfully to the sleeping time ($T_{Sleep})$. DC can be expressed as [39]:

(31) $$DC_{CD-ICC}=\frac{T_{C}+T_{suc}+T_{fail}}{T_{Sleep}}\;\left(1+PER_{CD-ICC}\right)$$

The packet error rate (PER) of each link is defined as the probability which at least one bit in a packet is erroneous and can be expressed as:

(32) $$ER=1-{\left(1-P_{b}\right)}^{S}$$

where, $P_{b}$ is the bit error rate (BER) and S is the payload size, and it set to ‘1’. In this paper, the DPSK modulation is utilized and $P_{b}$ expressed as:

(33) $$P_{b}=Q\;\left(2\gamma_{i,j}\;{\left|a_{i,j}\right|}^{2}\;10^{\sigma_{ij}}\right)≅\frac{1}{2}\exp\left(\gamma_{i,j}\cdot d_{ij}^{-\rho }\;10^{\sigma_{ij}}\right)$$

where, $\gamma_{i,j}$ is the signal to interference and noise ratio between two nodes. The $P_{b}^{CD-ICC}$ of the proposed protocol can be expressed as:

(34) $$P_{b}^{CD-ICC}=P_{\xi }\left(\xi_{thd}\right)\;P_{b}^{ICC}+\left(1-P_{\xi }\left(\xi_{thd}\right)\right)P_{b}^{sd}$$

where, $P_{b}^{ICC}$ is the probability of the ICC and can be expressed as:

(35) $$P_{b}^{ICC}=P_{b}^{sd}\left(1-P_{b}^{sr}\right)+\left(1-P_{b}^{sd}\right)P_{b}^{sr}\;P_{b}^{rd}$$

where, $P_{b}^{sd},\;P_{b}^{sr}$ and $P_{b}^{rd}$ are bit error probability of S−D, S−R and R−D links. Insert (20), (25), (29) and (34) in (31), we obtain the duty cycle of the proposed protocol.

The complete average power transmission, $P_{av},$ can be obtained by multiplying DC, $V_{dd}$, and $I_{act}$ where $V_{dd}$ is the radio frequency (RF) of the module supply voltage, and $I_{act}$ is the average RF active average current in a one-time frame [39]:

(36) $$P_{av}=DC_{CD-ICC}\;V_{dd}\;I_{act}$$

## 6. Simulation and Results Discussion 

In this section, the performance of the CD-ICC protocol that is presented in the aforementioned sections has been evaluated in terms of successful transmission probability, e2e delay, duty cycles, and average power transmission. In the simulation, random topology has been considered, where sensors are randomly distributed in 3.5×3.5 square area with normalized distance, the number of sensors are fixed in this area. The destination is located at the origin (0, 0), and correspondence source sensor located at $\left(d_{sd},0\right)$, in addition, the number of relay sensors are varying and randomly deployed between source and destination. The SINR threshold $\gamma_{thd}$ is set to be 0 dB. The pseudo code of the CD-ICC based on IEEE 802.15.6 policy and numerical parameter used in this paper given in Algorithm 1 and Table 3, respectively.

**Algorithm 1:** CD-ICC Pseudo Code.**Require**: $d_{sd}=0.1:0.1:3.5$, $scal=\mathrm{random}\;\left(1,\;\mathrm{length}\left(d_{\mathrm{sd}}\right)\right)$, $\xi_{max}$, $\gamma_{thd}$, **01** **begin****02** $for\;d_{sd}$**03** $for\;z=1:1:\mathrm{length}\left(d_{\mathrm{sd}}\right)$**04** $d_{sr}=scal\;\left(z\right)*\;d_{sd}\left(z\right)$**05** $d_{rd}=scal\;\left(z\right)*d_{sd}\;\left(z\right)$**06** Select $\xi_{min}$ ∈ random [0, 7]**07** Calculate back-off time given in (27)**08** Calculate $R_{ate}$ given in (30)**09** Calculate time as**10** $T_{x}=\frac{number\;of\;bits\;of\;x}{R_{ate}}$**11** Calculate e2e delay given in (19)**12** Calculate duty cycle given in (31)**13** Calculate average power consumption given in (32)**14** **Endfor****15** **Endfor**

**Figure 3:**
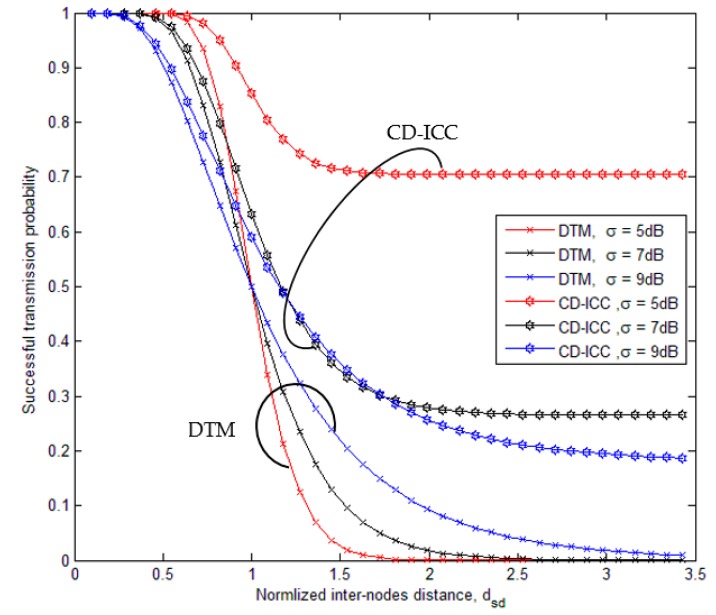
Comparison of successful transmission probability of DTM and CD-ICC with normalized inter-node distance, ρ is 3.5, $\xi_{min}$ is 6, and $\xi_{max}$ is 7.

Figure 3, shows the comparison of successful transmission probability of the DTM, and CD-ICC protocol as a function of $d_{sd}$ and σ. The important results appeared in the figure:

1. In the case of σ>0 dB the successful transmission probability is vary, which it is reduced at the short distances and increases at large distances this is due to signal fluctuations become more at σ>0 dB.
2. Even at large distance (greater than normalized threshold distance ‘1’), we get successful transmission probability less than 0.7.
3. The proposed protocol shows better successful transmission probability at the short and large distance compared to DTM.
4. For the low values of σ correspond to small variations of the signal power and high values of σ corresponding to stronger power variations.
5. At distance $d_{sd}$ = 2, and σ=9 dB, the successful transmission probability increased by 1.6 times over DTM. Further, At the distance $d_{sd}$ = 2, and σ=7 dB, the successful transmission probability increased by 5 times over DTM. While, At distance $d_{sd}$ = 1.5, anda σ=5 dB, the successful transmission probability increased by 13 times over DTM.

**Figure 4:**
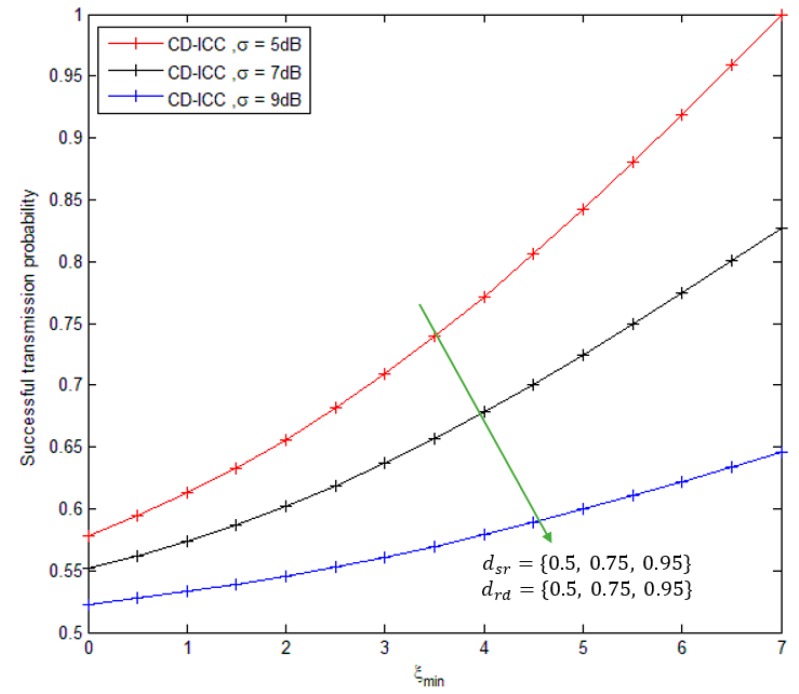
Successful transmission probability of CD-ICC with $\xi_{min}$. In all cases, $d_{sd}$ is 1, and ρ is 3.5.

Figure 4, shows the comparison of successful transmission probability of the CD-ICC protocol as a function of $\xi_{min}$. The important results appeared in the figure: As the $\xi_{min}$ increases, the successful transmission probability required is rises, in order to transmit the critical data efficiently. We can also note from Figure 3 that as the inter-nodes distance of S−R and R−D links are decreases, the successful transmission probability is increased.

**Figure 5:**
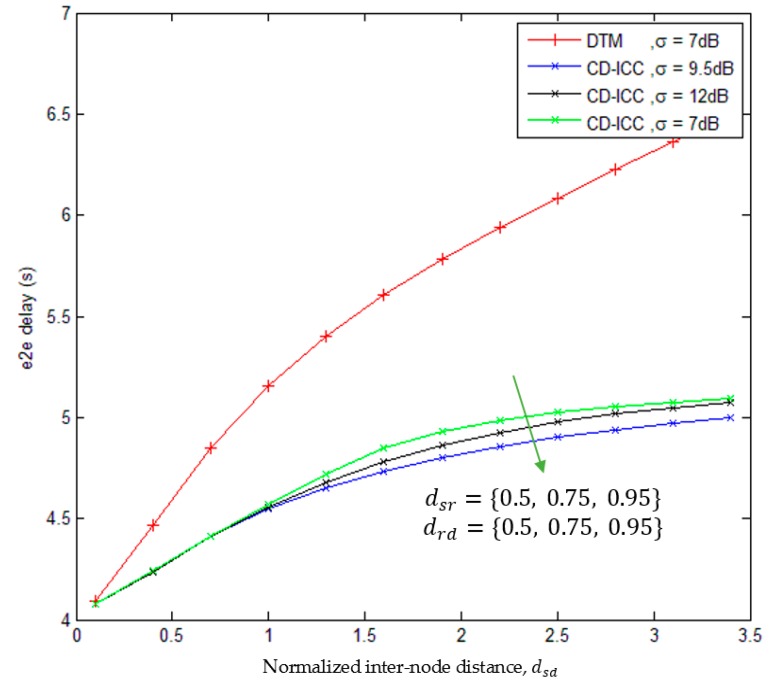
Comparison of e2e delay of DTM and CD-ICC with normalized inter-node distance, $d_{sd}$. In all cases, the ρ is 3.5, $\xi_{min}$ is 6 and $\xi_{max}$ is 7.

Figure 5, shows the comparison of end-to-end delay of the DTM, and CD-ICC protocol as a function of $d_{sd}$ and σ. The important results appeared in the figure:

1) The e2e delay of CD-ICC is less compared to the DTM.
2) For large distances between S−R and R−D links, the e2e delay is high.
3) At distance $d_{sd}$ = 2, and σ=12 dB, the e2e delay is reduce by 23.5% compared to DTM. Further, at distance $d_{sd}$ = 2, anσ=9.5 dB, the e2e delay is reduced by 20% compared to DTM. However, at distance $d_{sd}$ = 2, and σ=7 dB, the e2e delay is reduced by 18% compared to DTM.

**Figure 6:**
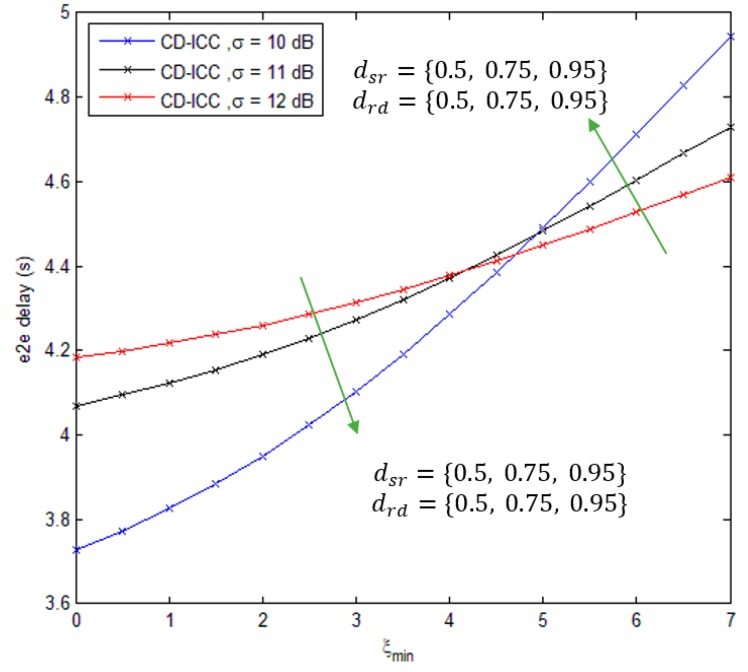
e2e delay of CD-ICC with $\xi_{min}$. In all cases, $d_{sd}$ is 1 and ρ is 3.5.

Figure 6, shows the comparison of e2e delay of CD-ICC protocol as a function of $\xi_{min}$. The important results appeared in the figure are summarized as follows: As the $\xi_{min}$ increases, e2e delay increases as well that is because of the critical data sent over different paths to guarantee delivering of the data to the destination. Furthermore, It can be seen from Figure 5 that at the large $\xi_{min}$ (more than 5), the e2e delay is large at small $d_{sr}$ and $d_{rd}$ and vice versa.

**Figure 7:**
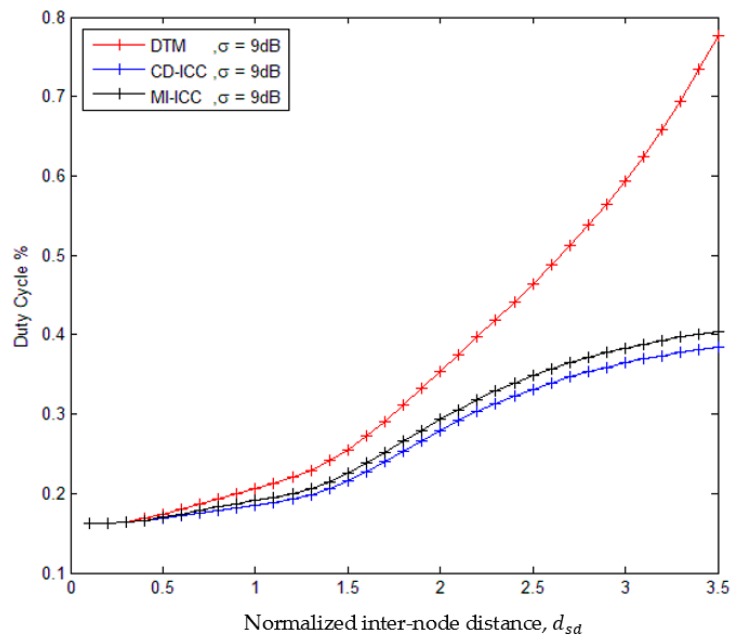
Comparison of Duty cycle of DTM, MI-ICC [34] and CD-ICC with normalized inter-node distance, $d_{sd}$. In all cases, the ρ is 3.5, $T_{sleep}$ is 25s, $\xi_{min}$ is 6 and $\xi_{max}$ is 7.

Figure 7 shows the comparison of the duty cycle of the DTM, MI-ICC [34], and CD-ICC protocol as a function of $d_{sd}$. The important results appeared in the figure: The duties cycle of the DTM, MI-CC and CD-ICC are reduced with large $d_{sd}$. While the duties cycle of the MI-CC and CD-ICC less than DTM. However, the duties cycle of CD-ICC is less than MI-ICC. At distance $d_{sd}$ = 2.5, the duty cycle of CD-ICC is improved by 60% compared to DTM and by 13% compared to MI-ICC. We consider MI-ICC because it support critical data, while CD-ICC support both critical and normal data. 

**Figure 8:**
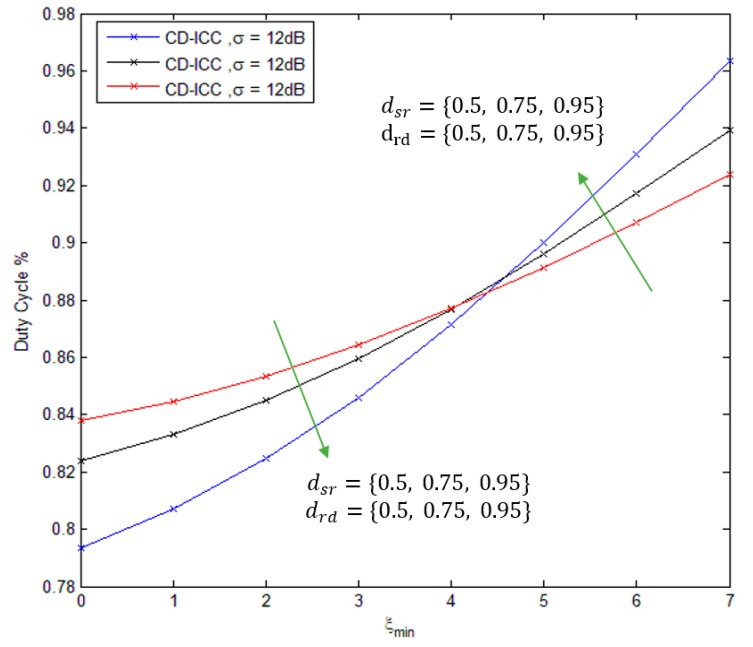
Duty cycle of CD-ICC with $\xi_{min}$. In all cases, $d_{sd}$ is 1, σ is 12 dB, $T_{sleep}$ is 40s and ρ is 3.

Figure 8, shows the comparison of the duty cycle of CD-ICC protocol as a function of $\xi_{min}$. Where, as the $\xi_{min}$ increases, duty cycle increases as well that is because of the critical data sent over different paths to guarantee data delivering. When the relay sensor located at mid-distance between source and destination, the duty cycle is reduced for $\xi_{min}$ (<5). On the other hand, the duty cycle is increased for $\xi_{min}$ (>5), when the relay sensor located far away from source and destination.

**Figure 9:**
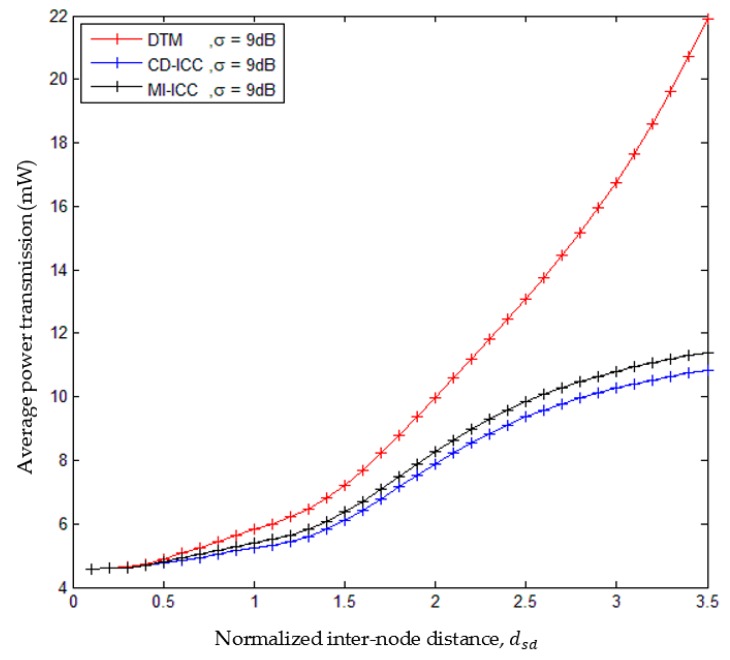
Comparison of average power transmission of DTM, MI-ICC and CD-ICC with normalized inter-node distance, $d_{sd}$.

**Figure 10:**
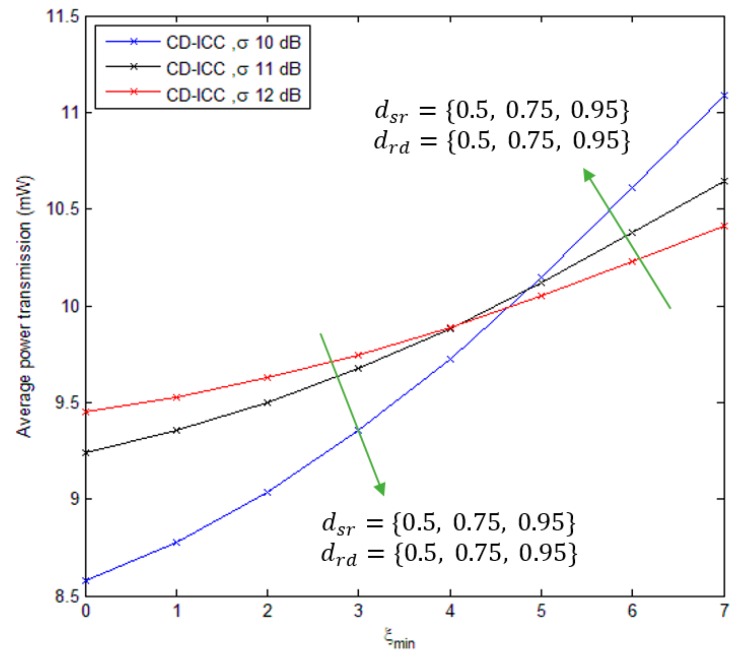
Average power transmission of CD-ICC with $\xi_{min}$. In all cases, $d_{sd}$ is 1, σ is 12 dB and ρ is 3.

Figure 9, shows the comparison of average power transmission of the DTM, MI-ICC [34] and CD-ICC protocol as a function of $d_{sd}$ and σ. The average power transmission of the proposed protocol is low compared to MI-ICC [34] and DTM. In addition, at distance $d_{sd}$ = 2.5, the power saving of CD-ICC with respect to DTM is 37.5%, and with respect to MI-ICC is 10%. Finally, Figure 10 shows the comparison of the average power transmission of CD-ICC protocol as a function of $\xi_{min}$. When the $\xi_{min}$ is growing up, then more power transmission is required to deliver the data efficiently to the destination.

## 7. Conclusions

In this paper, we have proposed a novel cooperative communication protocol for WBAN that is aware of the nature of the gathered data. It is based on the IEEE 802.15.6 CSMA policy under a lognormal shadowing channel model and is called CD-ICC. We have also proposed a new back-off procedure to be aware of the strategy for relay selection and chooses the best relay in an efficient and distributed manner. The proposed protocol increases the probability of a successful transmission if the gathered data were critical. In addition, we have demonstrated that the CD-ICC can substantially enhance the successful transmission, reduce e2e delay, and enhance power saving, compared to DTM IEEE 802.15.6 CSMA and MI-ICC. To this end, we have shown that the power saving of the CD-ICC is 37.5% with respect to DTM IEEE 802.15.6 CSMA and 10% with respect to MI-ICC. In future work, we will design and investigate a MAC protocol for inter-WBAN cooperation. 

## Figures and Tables

**Table 1:** Comparison of state of art work.

Pub. Year [Ref. No.]	Proposed Protocol	Metrics (Problem Addressed)	Compared with	Limitations
2013 [27]	ICC	Energy efficiency Optimal packet size	Single hop	MAC protocol not considered (IEEE 802.15.6). Duty cycle is not considered. e2e delay is not analysed. Best relay node selection is not considered. Nature of the gathered data is not considered.
2015 [28]	Packet size optimisation of ICC	Outage probability Successful transmission probability Energy efficiency	Single hop	MAC protocol not considered (IEEE 802.15.6) Duty cycle is not considered. e2e delay is not analysed. Best relay node selection is not considered Nature of the gathered data is not considered
2015 [29]	ICC	Throughput Average power consumption Propagation delay	Dual hops	MAC protocol not considered (IEEE 802.15.6) e2e delay is not analysed Best relay node selection is not considered It used TDMA which is unsuitable for WBAN Nature of the gathered data is not considered
2015 [30]	ICC	Packet error rate Energy efficiency	Single hop	MAC protocol not considered (IEEE 802.15.6) Duty cycle is not considered. e2e delay is not analysed Best relay node selection is not considered Nature of the gathered data is not considered
2015 [31]	Cooperative Energy Harvesting (CEH)-MAC	Network throughput Average e2e delay Energy efficiency	Single hop-IEEE 802.15.6 standard	e2e delay is not analysed Nature of the gathered data is not considered
2016 [32]	Incremental Cooperative Critical Data Transmission in Emergencies For Static WBAN (InCo-CEStat)	Reliability Residual energy increases. Throughput	Co-CEStat and EInCo-CEStat	MAC protocol not considered (IEEE 802.15.6) Duty cycle is not considered. The e2e delay is not analysed Best relay node selection is not considered
2016 [33]	Linear Acceleration based Transmission Power Decision Control (LA-TPDC)	Energy consumption, Signal-to-noise ratio (SNR), Bit error rate (BER), Packet delivery ratio (PDR)	TCC	MAC protocol not considered (IEEE 802.15.6) The e2e delay is not analysed Best relay node selection is not considered It used TDMA which is unsuitable for WBAN Support critical data
2018 [34]	A mutual information (MI)-based ICC	Network life time Residual energy Number of packets transmitted	Two-relay based, and ICC	MAC protocol not considered (IEEE 802.15.6) Duty cycle is not considered. The e2e delay is not analyse Support critical data

**Table 2:** Threshold and probability of the Critical Data Index.

$\xi_{min}\;$	$\xi_{max}$	$\xi_{thd}$	$P\;\left(\xi \geq \xi_{thd}\right)$
0	7	1	0.15
1	7	0.857	0.2255
2	7	0.714	0.3126
3	7	0.571	0.4194
4	7	0.428	0.540
5	7	0.285	0.6869
6	7	0.142	0.8408
7	7	0.0	1.0

**Table 3:** Numerical parameters.

Frequency band [MHz]	402–405 (MICS)
Bandwidth [MHz]	1
Maximum transmission rate (R) [Kbps]	75.9
Threshold transmission rate ($\beta_{o}$) [Mbps]	1
Modulation	DPSK
Payload size [bits]	2000
Minimum contention windows *CW*min [slots]	16
Maximum contention windows *CW*max [slots]	64
SINR threshold ($\gamma_{thd}$) [dB]	0
MAC header [bits]	56
MAC footer [bits]	16
PHY header [bits]	32
RF transceiver power on ($T_{on}$) [s]	2
Short interframe spacing time TpSIFS [µs]	50
Preamble [bits]	88
Slot time Ts [µs]	125
Delay time α [µs]	1
Maximum critical data index $\xi_{max}$	7
